# A Mechanistic Dissection of Multifaceted Complement Attack in Age‐Related Macular Degeneration Reveals the Superiority of C3 Inhibition

**DOI:** 10.1002/advs.78539

**Published:** 2026-10-28

**Authors:** Zhidong Chen, Mingshuang Wang, Huiqin Liu, Feng Qian

**Affiliations:** ^1^ School of Pharmaceutical Sciences Beijing Visual Science and Translational Eye Research Institute (BERI) Tsinghua Medicine Beijing Frontier Research Center for Biological Structure Tsinghua University Beijing People's Republic of China; ^2^ Quaerite Biopharm Research Beijing People's Republic of China

**Keywords:** age‐related macular degeneration (AMD), complement C3, complement C5, geographic Atrophy (GA), retinal pigment epithelium (RPE)

## Abstract

Age‐related macular degeneration (AMD) is the leading cause of irreversible central vision loss in the elderly, and geographic atrophy (GA) represents its advanced atrophic form. While C5 and C3 inhibitors have shown efficacy in slowing GA progression, the precise pathogenic roles of individual complement effectors and the optimal inhibition strategy remain subjects of ongoing debate. This study employs an in vitro model using A2E‐laden retinal pigment epithelium (RPE) cells and normal human serum to systematically investigate complement‐mediated RPE degeneration and compare the protective effects of different complement‐targeting strategies. We demonstrate that C5 blockade, while preventing lytic cell death caused by the membrane attack complex (MAC), fails to fully restore mitochondrial function or prevent phagolysosomal damage. Crucially, these C5‐independent pathologies involve C3 activation products. In contrast, upstream C3 inhibition comprehensively protects RPE cells by ameliorating these diverse pathological processes. Our findings provide a detailed mechanistic understanding of complement‐driven RPE degeneration and support a mechanistic rationale for an upstream C3‐targeting strategy in GA secondary to AMD.

## Graphical abstract

Complement‐mediated RPE degeneration extends beyond terminal MAC injury. A four‐hit mechanism emerges in which C3a and C5a disrupt mitochondrial function, while C3b and MAC compromise the phagolysosomal system. Upstream C3 inhibition suppresses this full spectrum of pathogenic effectors, providing broader cellular protection than C5 blockade.

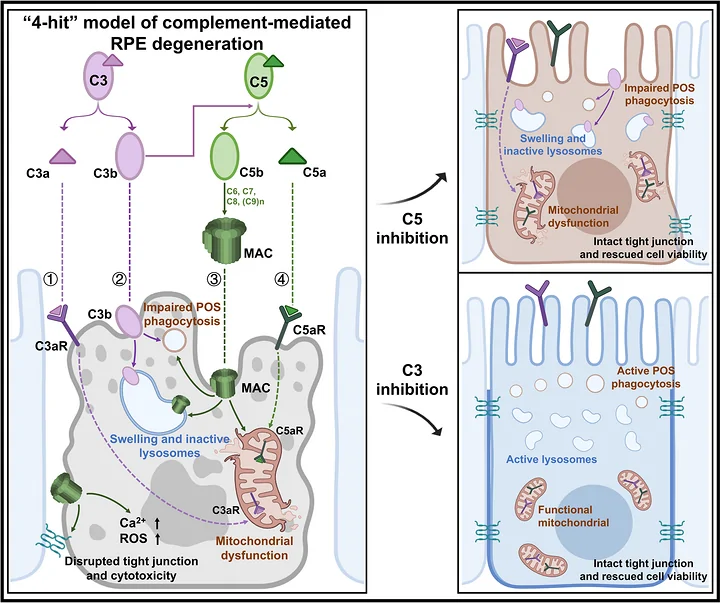

## 1 Introduction

Age‐related macular degeneration (AMD) is the leading cause of irreversible central vision loss in the elderly [1, 2]. Advanced AMD comprises two major clinical phenotypes: neovascular AMD (nAMD), characterized by macular neovascularization, and geographic atrophy (GA), the advanced form of non‐neovascular/atrophic AMD [1, 2]. Characterized by progressive degeneration and loss of the retinal pigment epithelium (RPE), photoreceptors, and choriocapillaris, GA is a multifactorial disease arising from a complex interplay between aging, genetic susceptibility, and environmental risk factors [1]. Given the central role of RPE degeneration in GA progression, elucidating the mechanisms underlying RPE injury is essential for developing effective therapeutic strategies.

Currently, substantial evidence has identified dysregulation of the complement system as a central pathogenic driver of AMD and GA [1, 3]. Moreover, the FDA approval of the C3 inhibitor Syfovre (pegcetacoplan) [4] and C5 inhibitor Izervay (avacincaptad pegol) [5] for GA validates the complement cascade as a therapeutic target [6]. Although targeting the complement system represents an attractive drug development strategy, it remains unclear which specific pathway and complement protein should be targeted for the optimal therapeutic outcome [3]. In the complement cascade, three activation pathways, classical pathway (CP), lectin pathway (LP), and alternative pathway (AP), converge on the cleavage of C3, which in turn leads to the cleavage of C5 and the subsequent formation of the pro‐inflammatory anaphylatoxin C5a and the lytic C5b9 membrane attack complex (MAC). The CP and LP are poised for complement cascade initiation in the retina of patients, due to elevated levels of their activators (e.g., carboxyethyl pyrrole autoantibodies [7], C‐reactive protein [8], and mannose‐binding lectin [9]), and the AP amplification loop serves as the dominant catalytic mechanism driving excessive complement activation and retinal cellular damage [10, 11].

Given their central role in the convergence of all three complement activation pathways, C3 and C5 are functionally convergent nodes and ideal targets for achieving comprehensive suppression of the complement system [3]. Both C5 inhibitor Izervay and C3 inhibitor Syfovre have demonstrated efficacy in slowing the progression of GA lesions in large‐scale clinical trials, while the relative efficacy of the C3 inhibitor and C5 inhibitor cannot be confirmed due to the lack of head‐to‐head comparisons [12, 13]. Other complement‐targeting strategies, such as targeting the terminal pathway by CD59 [14], blocking AP by complement factor [15], or inhibiting CP via anti‐C1q antibody [16], are currently under clinical evaluation. This diverse therapeutic landscape suggests that there is no clear consensus within the field regarding the relative contribution of each complement component or which complement protein represents the optimal therapeutic target.

Several previous studies have reported the pathological effects of individual complement components in RPE degeneration, including the dysregulated secretion of growth factors triggered by exogenous C3a and C5a [17, 18], drusen formation and metabolic dysfunction triggered by exogenous C3a [19, 20], mitochondrial abnormalities and lytic effects induced by persistent MAC [21, 22]. While these separate studies suggest that different complement proteins exert diverse effects on RPE cells, there is a lack of systematic comparative analysis to investigate the relative contributions of individual components within the intact complement system, specifically one that considers both physiological concentrations of constituents and their cascade interactions.

To address this, in the present study, we utilized normal human serum (NHS) to introduce the intact complement system and systematically investigate the damaging effects of different complement components. To investigate complement‐mediated RPE degeneration associated with advanced AMD, we established an A2E‐laden RPE model that reproduces several pathological features relevant to aging and AMD according to previous reports [23]. Briefly, RPE cells were laden with the cytotoxic bisretinoid N‐retinylidene‐N‐retinylethanolamine (A2E), a major component of lipofuscin that accumulates in aging or diseased RPE [24, 25, 26]. A2E accumulation has been reported to impair the trafficking of CD59 to the cell surface, compromising the cell's intrinsic defense against MAC formation and rendering it more susceptible to complement‐mediated damage [23, 27]. To characterize the multifaceted pathology of RPE cells after complement activation, a comprehensive spectrum of key RPE functions was investigated in this study, including cell viability, the integrity of the epithelial barrier, lysosomal and mitochondrial function, and photoreceptor outer segment (POS) phagocytosis capacity. A systematic, head‐to‐head comparison of different complement inhibition strategies was carried out to delineate the relative contributions of key complement components and validate their potential as therapeutic strategies.

Here, we demonstrate that C5 inhibition can protect RPE from MAC‐dependent lytic injury but can only partially rescue the non‐lytic functional damage in RPE induced by complement activation. The combination of a C3a receptor (C3aR) antagonist and C5 blocker can fully restore mitochondrial function but can only partially protect the phagolysosomal function. Only upstream C3 blockade, which prevents the generation of C3 cleavage products and downstream complement effectors, restored the full spectrum of measured cellular phenotypes, thereby conferring comprehensive protection to RPE cells. This study provides critical mechanistic insights into complement‐mediated RPE degeneration and provides a mechanistic rationale for upstream C3 inhibition in GA secondary to AMD.

## 2 Results

### 2.1 C5 Inhibition Protects RPE Cells From MAC‐Dependent Lytic Injury

To introduce the intact complement system, 40% NHS was used in our study, which exhibited high hemolytic activity indicative of classic complement activation and MAC formation. In contrast, 40% heat‐inactivated NHS (HI‐NHS) had no significant hemolytic effect and was used as a negative control (Figure S1).

To elucidate the mechanisms of complement‐mediated degeneration, we employed a pathologically relevant model of GA in which RPE cells were laden with lipofuscin A2E, mimicking a key pathological feature of aging and GA. Consistent with previous research [23], we demonstrated that A2E loading (Figure S2A) led to a downregulation of key complement regulatory proteins CD46, CD55, and CD59 (Figure S2B,C) on the cell surface, which is also observed in GA patients [28]. This loss of intrinsic protection led to excessive complement activation upon NHS treatment, characterized by significant deposition of C5b9 and C3b on the RPE cell surface (Figure 1A,B, and Figure S2D), and increased levels of C3a and C5a in culture supernatant (Figure S2E,F). This unchecked complement activation triggered a canonical cascade of acute cellular injury, beginning with an influx of extracellular calcium (Ca^2+^) (Figure 1C). As a critical second messenger, this sustained, uncontrolled Ca^2+^ influx is known to trigger aberrant intracellular signaling cascades that promote a pro‐inflammatory state and cellular stress [23, 29]. After complement activation, we also observed excessive reactive oxygen species (ROS) in A2E‐laden RPE cells (Figure 1D). Complement activation was also accompanied by prominent cytotoxicity, evidenced by a marked reduction in relative cell viability determined by the CCK‐8 assay (Figure 1E), increased membrane leakage detected by increased lactate dehydrogenase (LDH) release in culture supernatant (Figure S3A), and decreased intracellular ATP level (Figure S3B). Immunofluorescence staining for active caspase‐3, a marker of apoptosis, and phosphorylated receptor‐interacting protein kinase 3 (phospho‐RIP3), a marker of necrosis, indicated that cytotoxicity occurred via a mixture of apoptotic and necroptotic pathways (Figure S3C–E). Beyond direct cytotoxicity, complement activation also compromised the integrity of the RPE monolayer, causing a decrease in trans‐epithelial electrical resistance (TEER) and disorganization of the tight junction protein zonula occludens‐1 (ZO‐1), indicative of a disrupted outer blood‐retina barrier (Figure 1F,G).

**FIGURE 1:**
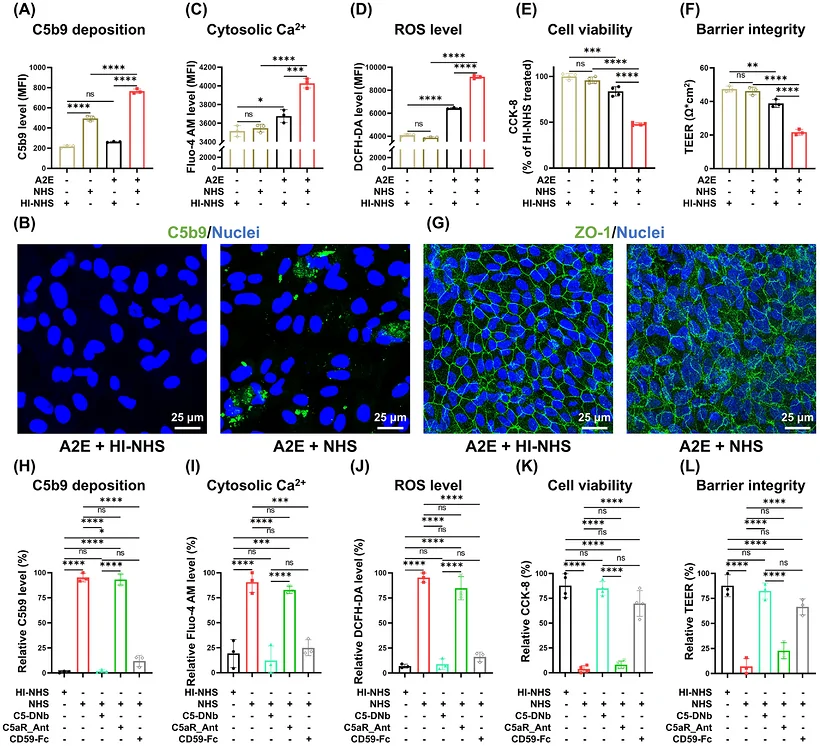
C5 inhibition protects A2E‐laden RPE cells from MAC‐dependent lytic injury. A2E‐laden ARPE‐19 cells were treated with 40% NHS or HI‐NHS. (A, B) C5b9 deposition was assessed by (A) flow cytometry, quantified as median fluorescence intensity (MFI), and (B) representative images of immunofluorescence (IF) staining of C5b9 deposition (green) in A2E‐laden RPE cells. (C) Cytosolic Ca^2+^ level was measured using the Fluo‐4 AM probe by flow cytometry and quantified as MFI. (D) Intracellular reactive oxygen species (ROS) level was measured using the DCFH‐DA probe by flow cytometry and quantified as MFI. (E) Cell viability was determined using the CCK‐8 assay and expressed as relative viability (%). Values were normalized to the HI‐NHS‐treated RPE cells without A2E loading, which served as the 100% viability reference. Data are presented as normalized relative values calculated as: Relative Viability (%) = [(OD450_sample_/OD450_HI‐NHS_)] × 100. (F, G) Epithelial barrier integrity was assessed in ARPE‐19 cultured on Transwell inserts. (F) Cell barrier integrity was assessed by Trans‐Epithelial Electrical Resistance (TEER), expressed as Ω*cm^2^. (G) Representative images of IF staining of the tight junction protein ZO‐1 (green) in A2E‐laden RPE cells. In (B, G), nuclei were counterstained with DAPI (blue). (H–L) Parallel assessment of A2E‐laden ARPE‐19 cells treated with NHS in the presence of C5‐DNb (20 µM), a C5aR antagonist (C5aR_Ant, Avacopan, 10 µM), or CD59‐Fc fusion protein (40 µM). Assays shown are (H) C5b9 deposition assessed by flow cytometry and quantified as MFI, (I) Cytosolic Ca^2+^ level measured using the Fluo‐4 AM probe by flow cytometry and quantified as MFI, (J) ROS level measured using the DCFH‐DA probe by flow cytometry and quantified as MFI, (K) Cell viability assessed by CCK‐8 assay and quantified by OD450, and (L) Epithelial barrier integrity assessed by TEER. In (H–L), data are presented as normalized relative values calculated as: Relative Value (%) = [(Sample − Min)/(Max − Min)] × 100. Data are presented as mean ± standard error of the mean (SEM) from *n* = 3 to 4 independent experiments. Statistical analyses were performed using GraphPad Prism 9 (https://www.graphpad.com) by one‐way one‐way analysis of variance (ANOVA) with Tukey's multiple comparison test. ^∗∗∗∗^ *p* < 0.0001, ^∗∗∗^ *p* < 0.001, ^∗∗^ *p* < 0.01, ^∗^ *p* < 0.05, ns = non‐significant.

To investigate the protective effect of terminal pathway inhibition, we employed C5‐DNb, a biparatopic anti‐C5 fusion protein containing two nanobody domains that bind distinct, non‐overlapping epitopes on C5. Simultaneous inhibition of two distinct C5 epitopes has been shown to achieve more complete inhibition of C5 activation and terminal pathway activity than single‐epitope C5 inhibitors [30, 31]. Consistent with this mechanism, C5‐DNb exhibited greater complement inhibition activity than parental single nanobody or the clinically approved single‐epitope C5 inhibitor avacincaptad pegol in the hemolysis assay (Figure S4). Consistently, C5‐DNb completely inhibited the deposition of C5b9 (Figure 1H), and all downstream manifestations of lytic injury were restored to baseline levels (Figure 1I–L). By inhibiting the deposition of MAC on the RPE cell surface, C5 blockade normalized intracellular Ca^2+^ levels (Figure 1I) and suppressed excessive ROS production (Figure 1J). In addition, C5 blockade also restored cell viability (Figure 1K) and barrier function of the RPE monolayer (Figure 1L).

To confirm that the above effects were mediated by the functional MAC or C5a‐C5aR signaling, we performed parallel experiments using an Fc‐fusion CD59 protein, which inhibits MAC assembly [32, 33], and a C5a receptor (C5aR) antagonist (Avacopan) [34], respectively. The Fc‐fusion CD59 protein partially recapitulated the protective effects of C5‐DNb, attenuating the complement‐induced Ca^2+^ influx, ROS accumulation, loss of cell viability, and TEER reduction (Figure 1I–L). Notably, unlike C5‐DNb, Fc‐fusion CD59 did not completely inhibit the C5b9 deposition signal (Figure 1H). This observation is consistent with the mechanism of CD59, which inhibits MAC assembly by preventing efficient C9 incorporation and polymerization rather than by blocking upstream C5 activation or the initial formation of terminal complement complexes [32]. Consequently, terminal complement complexes containing C9 may still be detected by the antibody aE11 despite being unable to form fully functional lytic pores [35]. In contrast, the C5aR antagonist did not influence the MAC deposition (Figure 1H) or significantly affect these readouts (Figure 1I–L). Together with the complete protection afforded by upstream C5 inhibition, these findings support a role for MAC in complement‐mediated lytic injury.

To directly verify the pathological activities of MAC, we treated A2E‐laden RPE cells with assembled MAC under serum‐free conditions (Figure S5). MAC was assembled by sequential addition of purified C5b6, C7, C8, and C9 proteins, and cells receiving C5b6 and C7 were set as a control (Figure S5A). Assembled MAC was confirmed by C5b9 deposition, and the degeneration phenotypes were observed following MAC treatment, including Ca^2+^ influx, ROS accumulation, and membrane leakage (Figure S5B–D). These findings demonstrate that MAC can reproduce the lytic injury induced by complement activation independently of other complement effectors.

### 2.2 C5 Inhibition can Only Partially Restore the Mitochondrial, Lysosomal, and Phagocytic Functions

Beyond cytotoxicity, we found that complement activation induced cellular stress in A2E‐laden RPE cells, such as Ca^2+^ influx (Figure 1C) and ROS accumulation (Figure 1D). These effects are known as stressors that can lead to organelle damage, such as mitochondrial and lysosomal dysfunction. Therefore, we investigated in greater detail whether C5 blockade can rescue RPE cells from a broad range of fundamental organellar dysfunctions and restore overall cellular homeostasis.

Indeed, complement activation induced significant organellar damage in A2E‐laden RPE cells. Mitochondrial integrity was compromised, evidenced by a marked decrease in mitochondrial membrane potential measured by tetramethylrhodamine ethyl ester (TMRE) (Figure 2A,B) and reduced intensity of the mitochondrial membrane protein translocase of the outer mitochondrial membrane 20 (TOMM20) (Figure 2C,D). The structure and function of lysosomes, which are critical for cellular clearance, were also severely damaged, showing reduced lysosome‐associated membrane protein 2 (LAMP2) abundance (Figure 2E,F), fewer acidic lysosomal compartments detected by LysoTracker Green DND‐26 (Figure 2G,H), and fewer cathepsin D‐positive puncta (Figure 2I,J). The POS phagocytic capacity was also impaired (Figure 2K,L).

**FIGURE 2:**
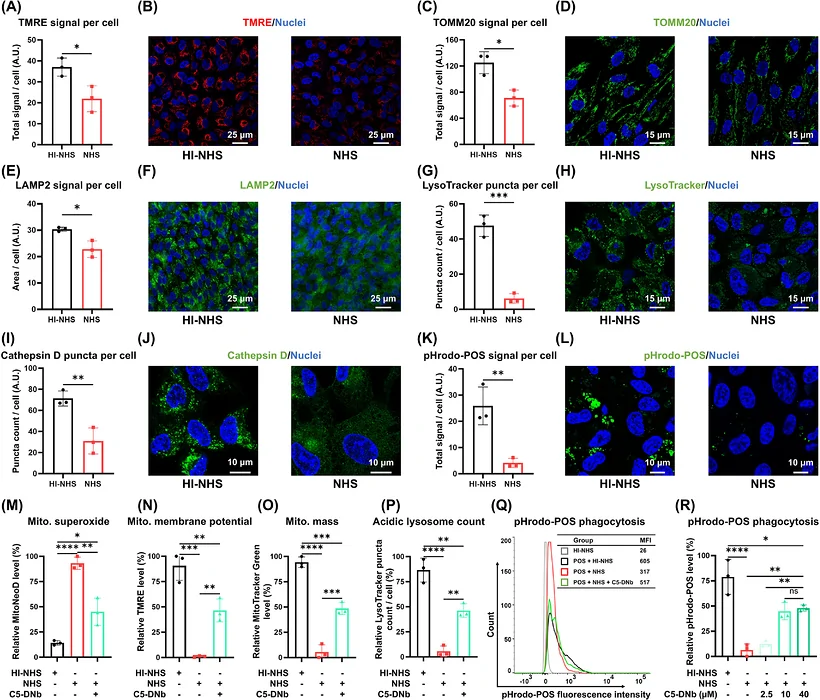
C5 inhibition only partially restores mitochondrial, lysosomal, and phagocytic functions. (A–L) Characterization of organellar morphology and function in A2E‐laden ARPE‐19 cells treated with NHS or HI‐NHS. (A, B) Mitochondrial membrane potential was assessed by tetramethylrhodamine, ethyl ester (TMRE) staining (red), analyzed by IF staining and quantified as “MFI x total spot area/cell number”, referred to as “Total signals/cell”. (C, D) Mitochondrial mass was assessed by IF staining for the outer mitochondrial membrane protein TOMM20 (green) and quantified as “MFI x total spot area/cell number”, referred to as “Total signals/cell”. (E, F) Quantification of LAMP2 fluorescence intensity by IF staining and quantified as “MFI x total spot area/cell number”, referred to as “Total signals/cell”. (G, H) Acidic lysosomal compartments assessed by LysoTracker Green DND‐26 staining (green), analyzed by quantification of puncta number per cell, referred to as “Puncta count/cell”. (I, J) Lysosomal digestive enzyme level assessed by IF staining for Cathepsin D (green), analyzed by quantification of puncta number per cell, referred to as “Puncta count/cell”. (K, L) Phagocytic capacity was assessed by uptake of pHrodo‐labelled bovine POS (green), quantified as “MFI x total spot area/cell number”, referred to as “Total signals/cell”. In (B, D, F, H, J, L), nuclei were counterstained with Hoechst (blue) or DAPI (blue). (M–P) Assessment of organellar dysfunction in cells treated with HI‐NHS, NHS, or NHS in the presence of C5‐DNb (20 µM). (M) Mitochondrial superoxide level was measured using the MitoNeoD probe by flow cytometry and quantified as MFI. (N) Mitochondrial membrane potential was measured using TMRE by flow cytometry and quantified as MFI. (O) Mitochondrial mass was measured using the MitoTracker Green FM by flow cytometry and quantified as MFI. (P) Acidic lysosomal compartments were measured by IF staining for LysoTracker Green DND‐26, analyzed by quantification of puncta number per cell. (Q) POS phagocytic function was assessed by pHrodo‐POS signal and analyzed by flow cytometry, and quantified as MFI. (R) POS phagocytic function by pHrodo‐POS signal in cells treated with HI‐NHS, NHS, or NHS in the presence of C5‐DNb (2.5, 10, and 40 µM), analyzed by flow cytometry and quantified as MFI. In (M–P), data are presented as normalized relative values calculated as: Relative Value (%) = [(Sample − Min)/(Max − Min)] × 100. Data are presented as mean ± SEM from *n* = 3 independent experiments. Statistical analyses were performed using GraphPad Prism 9 (https://www.graphpad.com) by unpaired t‐test or one‐way ANOVA with Tukey's multiple comparison test. ^∗∗∗∗^ *p* < 0.0001, ^∗∗∗^ *p* < 0.001, ^∗∗^ *p* < 0.01, ^∗^ *p* < 0.05, ns = non‐significant.

Under the C5 blockade with C5‐DNb, mitochondrial superoxide level in A2E‐laden RPE cells remained pathologically elevated, and the mitochondrial membrane potential and mass failed to fully recover (Figure 2M–O). Similarly, acid lysosomal compartments remained impaired under C5 inhibition (Figure 2P). C5‐DNb produced only a 20%–60% recovery across mitochondrial and lysosomal readouts. In addition, C5 inhibition failed to fully rescue the capacity of POS phagocytosis, which relies on both mitochondrial integrity and lysosomal digestive activity (Figure 2Q). Critically, this incomplete protection was not a matter of insufficient inhibitor concentration. As shown in dose‐response analysis (Figure 2R), increasing C5‐DNb concentration up to 40 µM, supramaximal for completely inhibiting hemolysis, yielded no additional benefit in restoring phagocytic function. Collectively, this incomplete protection reveals the existence of significant C5‐independent pathogenic pathways that continue to drive cellular dysfunction even when the terminal lytic pathway is completely blocked.

### 2.3 Residual RPE Mitochondrial Dysfunction Under C5 Inhibition is Further Rescued by C3a Blockade

The persistence of cellular dysfunction under C5‐DNb pointed to the involvement of upstream complement effectors. Therefore, we next investigated the pathogenic roles of C3 activation products that persist under C5 blockade.

We first sought to determine whether C3a itself is sufficient to induce mitochondrial injury. Consistent with earlier reports [19], direct C3a treatment caused mitochondrial dysfunction in A2E‐laden RPE cells (Figure S6). These findings demonstrate that C3a alone is sufficient to induce mitochondrial injury independently of ongoing complement activation. Subsequently, we further evaluated the contribution of C3a signaling within the complement cascade to RPE cell injury by supplementing NHS with SB290157, a C3aR antagonist [36]. This blockade significantly mitigated the level of mitochondrial superoxide induced by NHS (Figure 3A). Recovery of mitochondrial membrane potential (Figure 3B) and mitochondrial mass (Figure 3C) was also observed upon SB290157 treatment. Furthermore, the cytoplasmic release of cytochrome c induced by NHS, which is an indicator of mitochondrial damage and apoptosis initiation and was observed by the increased staining area of cytochrome c, was partially suppressed by SB290157 (Figure 3D). These data identify the anaphylatoxin C3a as a key mediator of C5‐independent mitochondrial injury. More importantly, combined treatment with SB290157 and C5‐DNb resulted in a significantly greater rescue of mitochondrial function than either agent alone (Figure 3). The additive effect suggests that mitochondrial damage in RPE cells induced by the complement system involves both upstream product C3a and the activation of the terminal pathway.

**FIGURE 3:**
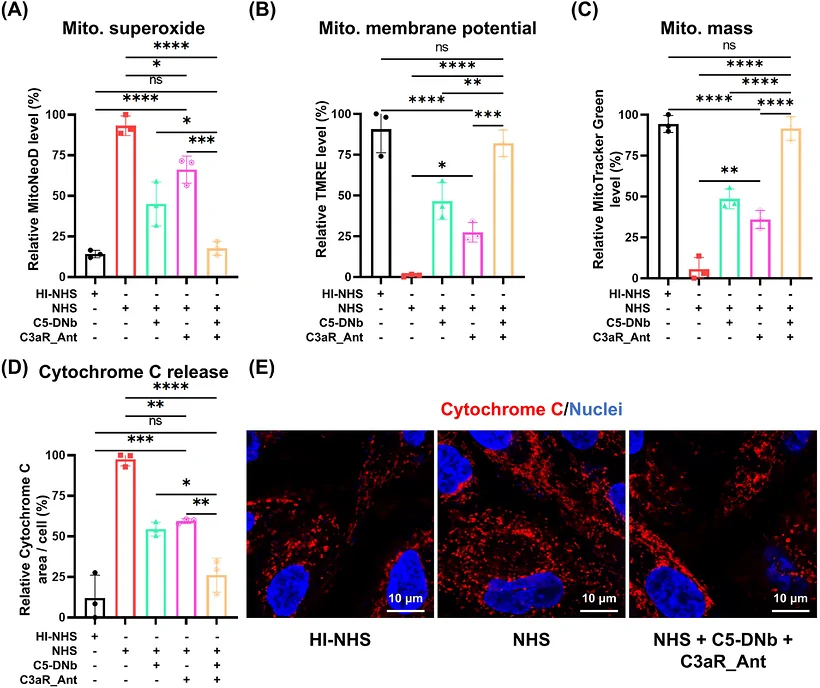
Residual RPE mitochondrial dysfunction under C5 inhibition is further rescued by C3a blockade. (A–E) A2E‐laden ARPE‐19 cells were treated with 40% NHS in the presence of C5‐DNb (20 µM) alone, the C3a receptor antagonist (C3aR_Ant, SB290157, 10 µM) alone, or the combination of both inhibitors. HI‐NHS served as the control. (A) Mitochondrial superoxide level was measured using the MitoNeoD probe by flow cytometry and quantified as MFI. (B) Mitochondrial membrane potential was measured using the TMRE by flow cytometry and quantified as MFI. (C) Mitochondrial mass was measured using MitoTracker Green FM by flow cytometry and quantified as MFI. (D) Cytochrome c release was assessed by IF staining for cytochrome c (red) and analyzed by quantification of cytochrome c area per cell. (E) Representative confocal images of cytochrome c staining in (D). Nuclei were counterstained with DAPI (blue). Data are presented as normalized relative values calculated as: Relative Value (%) = [(Sample − Min)/(Max − Min)] × 100. Data are presented as mean ± SEM from *n* = 3 independent experiments. Statistical analyses were performed using GraphPad Prism 9 (https://www.graphpad.com) by one‐way ANOVA with Tukey's multiple comparison test. ^∗∗∗∗^ *p* < 0.0001, ^∗∗∗^ *p* < 0.001, ^∗∗^ *p* < 0.01, ^∗^ *p* < 0.05, ns = non‐significant.

Next, we determined whether the contribution of terminal pathway activation to mitochondrial pathology was mediated by C5a, MAC, or both. We found that the anaphylatoxin C5a contributed to mitochondrial injury, as confirmed by direct C5a stimulation experiments and the partial rescue effect of the C5aR antagonist (Figure S6A–C). Moreover, the residual mitochondrial damage, which is not rescued by combined C3aR and C5aR antagonism (Figure S6A–C), was consistent with an additional contribution from MAC, since the combined treatment of SB290157 and C5‐DNb completely recovered these specific damage phenotypes (Figure 3). Consistent with this conclusion, partial protection by the CD59‐Fc further supports the contribution of MAC to mitochondrial dysfunction (Figure S6A–C). We further validated these findings using purified complement effectors in serum‐free conditions. C3a, C5a, and MAC each decreased mitochondrial membrane potential (Figure S6D) and mitochondrial mass (Figure S6E). In contrast, purified C3b did not significantly affect mitochondrial function. These direct experiments suggest that both anaphylatoxins and MAC contribute to mitochondrial dysfunction. Thus, C3a acts as a C5‐independent metabolic disruptor, causing the mitochondrial dysfunction that is further exacerbated by terminal pathway effectors, C5a and MAC.

### 2.4 C3b and MAC Contribute to Lysosomal and POS Phagocytic Dysfunction

After identifying the contribution of C3a and the terminal pathway to mitochondrial dysfunction, we next investigated the mechanisms underlying the persistent phagolysosomal dysfunction. The RPE is a phagocyte, tasked with the substantial daily burden of clearing shed POS to maintain retinal homeostasis [37]. Impairment of this process promotes the accumulation of cellular debris and contributes to RPE and photoreceptor degeneration in AMD.

We first examined whether the anaphylatoxins C3a and C5a contributed to these phenotypes. Neither the C3aR antagonist nor the C5aR antagonist significantly restored the number of LysoTracker‐positive acidic lysosomes (Figure 4A) or POS phagocytosis in NHS‐treated cells (Figure 4B). Furthermore, direct application of purified C3a or C5a to A2E‐laden RPE cells in 40% HI‐NHS did not significantly impair either lysosomal function or POS phagocytosis (Figure 4C,D). Therefore, these data from both blockade and direct stimulation approaches indicated that C3a and C5a are not direct drivers of the phagolysosomal dysfunction observed under these conditions, suggesting that other complement effectors contribute to phagolysosomal dysfunction.

**FIGURE 4:**
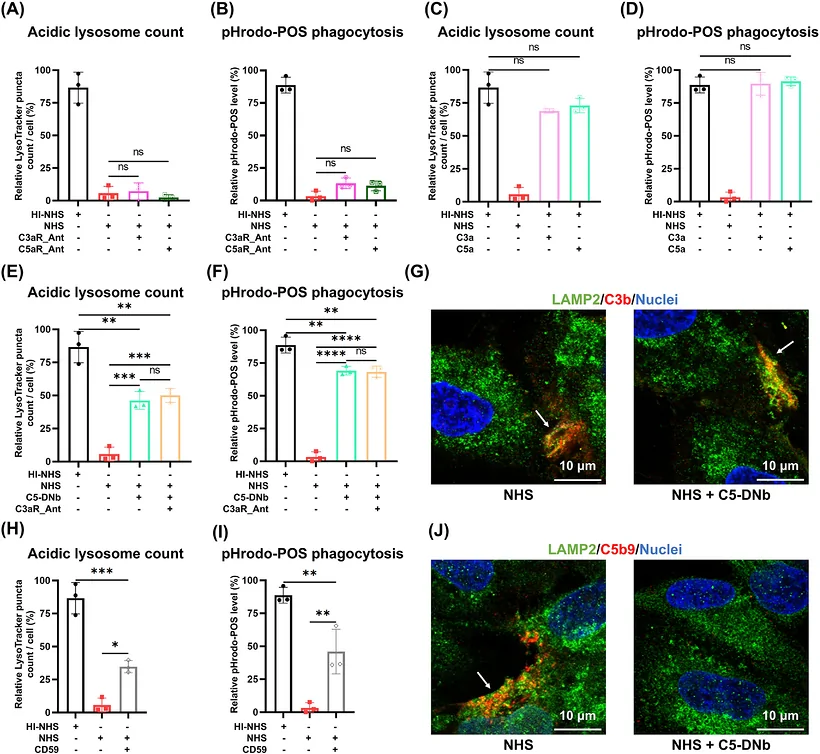
C3b and MAC contribute to lysosomal and POS phagocytic dysfunction. (A, B) A2E‐laden ARPE‐19 cells were treated with 40% NHS in the presence of the C3aR antagonist (SB290157, 10 µM) or C5aR antagonist (Avacopan, 10 µM). (A) Acidic lysosomal compartments measured by LysoTracker Green DND‐26 staining, analyzed by quantification of puncta number per cell. (B) POS phagocytic function assessed by pHrodo‐POS signal, analyzed by flow cytometry and quantified as MFI. (C, D) A2E‐laden ARPE‐19 cells were treated with 40% HI‐NHS in the presence of C3a protein (2 µg/mL) or C5a protein (2 µg/mL). (C) Acidic lysosomal compartments. (D) POS phagocytic function. (E, F) A2E‐laden ARPE‐19 cells were treated with 40% NHS in the presence of C5‐DNb (20 µM) alone or combination of C5‐DNb (20 µM) and the C3aR antagonist (SB290157, 10 µM). (E) Acidic lysosomal compartments. (F) POS phagocytic function. (H, I) A2E‐laden ARPE‐19 cells were treated with 40% NHS in the presence of CD59‐Fc fusion protein (40 µM). (H) Acidic lysosomal compartments. (I) POS phagocytic function. Data are presented as normalized relative values calculated as: Relative Value (%) = [(Sample − Min)/(Max − Min)] × 100. Data are presented as mean ± SEM from *n* = 3 independent experiments. (G, J) Confocal IF analysis of C3b and C5b9 signals within LAMP2‐positive lysosomal compartments. Cells were treated as indicated and co‐stained for lysosomal marker LAMP2 (green) and either (G) C3b (red) or (J) C5b9 (red). White arrows indicate co‐localization. In (G, J), nuclei were counterstained with DAPI (blue). Statistical analyses were performed using GraphPad Prism 9 (https://www.graphpad.com) by one‐way ANOVA with Tukey's multiple comparison test. ^∗∗∗∗^ *p* < 0.0001, ^∗∗∗^ *p* < 0.001, ^∗∗^ *p* < 0.01, ^∗^ *p* < 0.05, ns = non‐significant.

We next directly evaluated active complement effectors by applying purified C3a, C5a, or reconstituted MAC to A2E‐laden RPE cells under serum‐free conditions. Among the components tested, only MAC significantly reduced the number of LysoTracker‐positive acidic lysosomes and impaired POS phagocytosis (Figure S7).

Although MAC was sufficient to induce phagolysosomal dysfunction, it could not account for the persistent dysfunction after effective C5 inhibition, suggesting the involvement of additional complement mechanisms upstream of C5. Because C3b serves as an essential structural component of the C5 convertases, direct inhibition of C3b also affects downstream terminal pathway activation, and a selective experimental system to isolate the biological activity of C3b itself is currently not available. We therefore combined C5‐DNb with SB290157 to suppress C5 activation while inhibiting C3aR‐associated signaling, without preventing upstream C3 activation and C3b deposition. This combination provided no additional protection over C5‐DNb alone, and significant reductions in LysoTracker‐positive puncta and POS phagocytosis remained relative to the HI‐NHS control (Figure 4E,F). Because C3aR signaling and C5 activation were inhibited while upstream C3 activation and C3b deposition persisted, these findings implicated C3 activation products, particularly deposited C3b, in the residual phagolysosomal dysfunction. Consistent with this conclusion, C3b immunofluorescence signal was detected within LAMP2‐positive lysosomal compartments following NHS‐induced complement activation (Figure 4G, see also Figure S8 for unmerged individual channels). This compartmentalized signal indicates the presence of C3b within the endolysosomal system and may represent an additional burden on lysosomal processing. Together, these data implicate activation‐generated C3b in the residual C5‐independent phagolysosomal dysfunction.

Alongside this C3b‐associated mechanism, several complementary experiments support the contribution of MAC to phagolysosomal injury. In addition to the purified MAC treatment experiments (Figure S7), inhibition of MAC assembly by CD59‐Fc suggests the role of MAC (Figure 4H,I). Furthermore, C5b9 signal was detected within LAMP2‐positive lysosomal compartments (Figure 4J, see also Figure S8 for unmerged individual channels), suggesting that C5b9 within these compartments may also contribute to lysosomal burden following complement attack. Collectively, these findings support contributions from activation‐generated C3b‐containing products and MAC to phagolysosomal dysfunction.

### 2.5 Upstream C3 Inhibition Comprehensively Rescues RPE From the Complement‐Mediated Degeneration

The preceding findings reveal that the complement cascade inflicts damage through a complex interplay of effectors, C3a, C3b, C5a, and the MAC, each contributing to the degenerative phenotype. This led to a hypothesis that upstream inhibition of C3 represents a mechanistically broader strategy for RPE protection.

To validate this hypothesis, we systematically compared the comprehensive effects of both strategies on RPE survival and function. To evaluate the consequences of comprehensive upstream C3 complement inhibition, we employed the C3‐binding nanofitin (C3‐Nf) [38], which inhibits the generation of downstream complement effectors and has higher activity than Syfovre (pegcetacoplan) [39]. C3‐Nf inhibited the generation of all downstream effectors (C3a, C3b, C5a, and MAC) to levels indistinguishable from HI‐NHS controls, whereas C5‐DNb blocked only C5a and MAC generation, leaving C3a and C3b production intact (Figure S9).

The validity of this hypothesis was evident in two aspects. First, both strategies could prevent the acute, MAC‐dependent lytic assault. C3 inhibition normalized intracellular Ca^2+^ levels (Figure 5A) and suppressed excessive ROS production (Figure 5B). Cell viability (Figure 5C) and epithelial barrier integrity (Figure 5D) were also restored by C3‐Nf. The critical distinction lay in C5‐independent pathologies that the terminal blockade failed to address. Upstream C3 inhibition, by preventing the generation of downstream complement effectors, also resolved these non‐lytic cellular injuries. C3 blockade fully resolved the persistent mitochondrial dysfunction, normalizing all metrics of mitochondrial health, including superoxide level (Figure 5E), membrane potential (Figure 5F), mitochondrial mass (Figure 5G), and cytoplasmic release of cytochrome c (Figure 5H). Simultaneously, regarding the phagolysosomal axis, immunofluorescence analysis confirmed that C3 inhibition prevented the appearance of C3b‐ and C5b9‐associated signals within LAMP2‐positive lysosomal compartments (Figure 5I,J, see also Figure S8 for unmerged individual channels). This was accompanied by a complete functional rescue: C3 inhibition restored the number of acidic lysosomal compartments (Figure 5K) and normalized the POS phagocytic function (Figure 5L) to baseline levels. The broader protection afforded by C3‐Nf in phagolysosomal damage further supports a contribution of C3b to the residual phagolysosomal dysfunction under combined treatment of C5‐DNb and SB290157 (Figure 4E,F).

**FIGURE 5:**
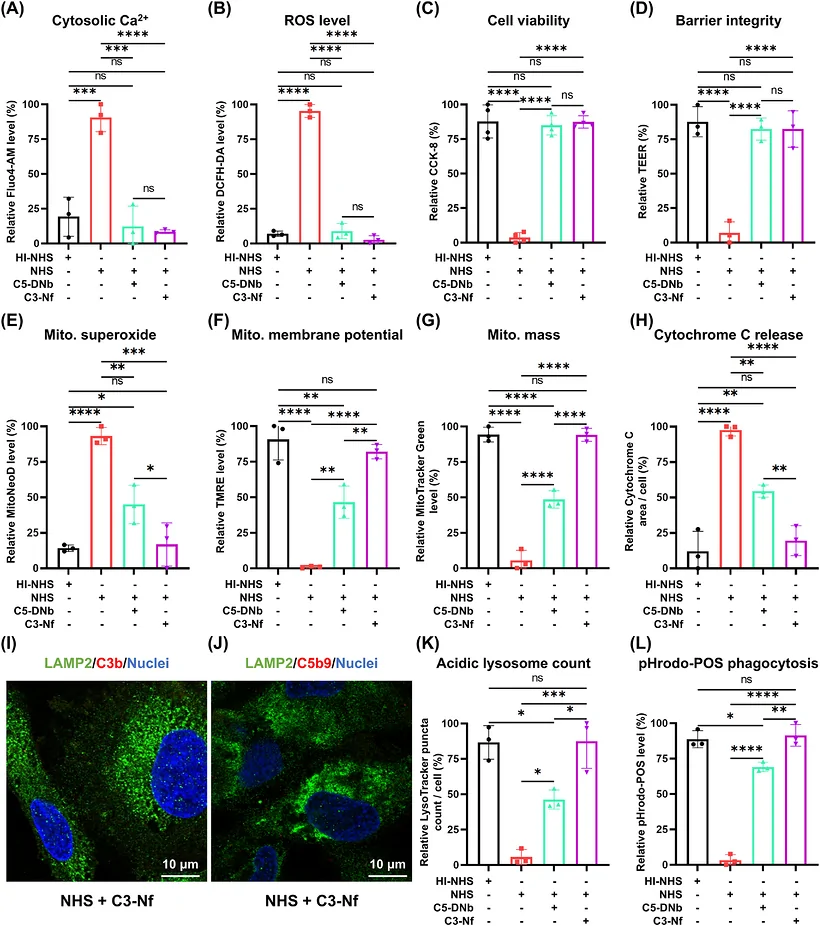
Upstream C3 inhibition comprehensively rescues RPE from complement‐mediated degeneration. A2E‐laden ARPE‐19 cells were treated with 40% NHS in the presence of either C5‐DNb (20 µM) or C3‐Nf (40 µM). HI‐NHS served as the control. (A) Cytosolic Ca^2+^ level was measured using Fluo‐4 AM by flow cytometry and quantified as MFI. (B) Intracellular ROS level was measured using DCFH‐DA by flow cytometry and quantified as MFI. (C) Cell viability was assessed by the CCK‐8 assay and quantified by OD450. (D) Epithelial barrier integrity was assessed by TEER measurement. (E) Mitochondrial superoxide level was measured using MitoNeoD by flow cytometry and quantified as MFI. (F) Mitochondrial membrane potential was measured using TMRE by flow cytometry and quantified as MFI. (G) Mitochondrial mass was measured using MitoTracker Green FM by flow cytometry and quantified as MFI. (H) Cytochrome c release was assessed by IF staining for cytochrome c and analyzed by quantification of cytochrome c area per cell. (I, J) Confocal IF analysis of C3b and C5b9 signals within LAMP2‐positive lysosomal compartments. Cells were treated as indicated and co‐stained for lysosomal marker LAMP2 (green) and either (I) C3b (red) or (J) C5b9 (red). The nuclei were counterstained with DAPI (blue). No detectable C3b or C5b9 signals were observed following C3 inhibition. (K) Acidic lysosomal compartments were measured by LysoTracker Green DND‐26 staining, analyzed by quantification of puncta number per cell. (L) POS phagocytic function was assessed by pHrodo‐POS signal, analyzed by flow cytometry and quantified as MFI. Data are presented as normalized relative values calculated as: Relative Value (%) = [(Sample − Min)/(Max − Min)] × 100. Data are presented as mean ± SEM from *n* = 3 to 4 independent experiments. Statistical analyses were performed using GraphPad Prism 9 (https://www.graphpad.com) by one‐way ANOVA with Tukey's multiple comparison test. ^∗∗∗∗^ *p* < 0.0001, ^∗∗∗^ *p* < 0.001, ^∗∗^ *p* < 0.01, ^∗^ *p* < 0.05, ns = non‐significant.

In summary, by inhibiting the broader spectrum of complement effectors at their source, C3 inhibition provides broader protection against the multifaceted complement‐mediated degeneration observed in RPE cells.

## 3 Discussion

This study was designed to dissect the multifaceted mechanisms of complement‐mediated RPE degeneration, considering both the physiological concentrations of complement constituents and their cascade interactions. In doing so, our results provide a mechanistic rationale for upstream C3 inhibition in GA secondary to AMD by a head‐to‐head comparison of different complement inhibition strategies. By systematically deconstructing the pathogenic cascade, our work provides several findings that deepen the understanding of the role of the complement system in RPE pathology. Specifically, we demonstrate that: (1) The conventional view focusing on MAC‐mediated lysis is incomplete. We deconstruct the intact complement cascade to reveal the different, non‐lytic, and significant contributions of upstream complement effectors, C3a and C3b, which drive profound cellular dysfunction under terminal pathway blockade; (2) The anaphylatoxin C3a is a C5‐independent driver of mitochondrial injury, alongside C5a and the MAC; (3) Activation‐generated C3b is implicated in phagolysosomal dysfunction in concert with MAC; (4) Upstream C3 inhibition is mechanistically superior to C5 blockade. The complement cascade inflicts RPE damage through a complex interplay of complement effectors, including C3a, C3b, C5a, and the MAC, each contributing to the degenerative cellular phenotype, forming a “4‐hit” mechanism of complement‐mediated RPE degeneration (Figure 6). These findings establish a clear mechanistic rationale for the superiority of C3 inhibition, as it is the only strategy that inhibits the full spectrum of pathogenic effectors at their source.

**FIGURE 6:**
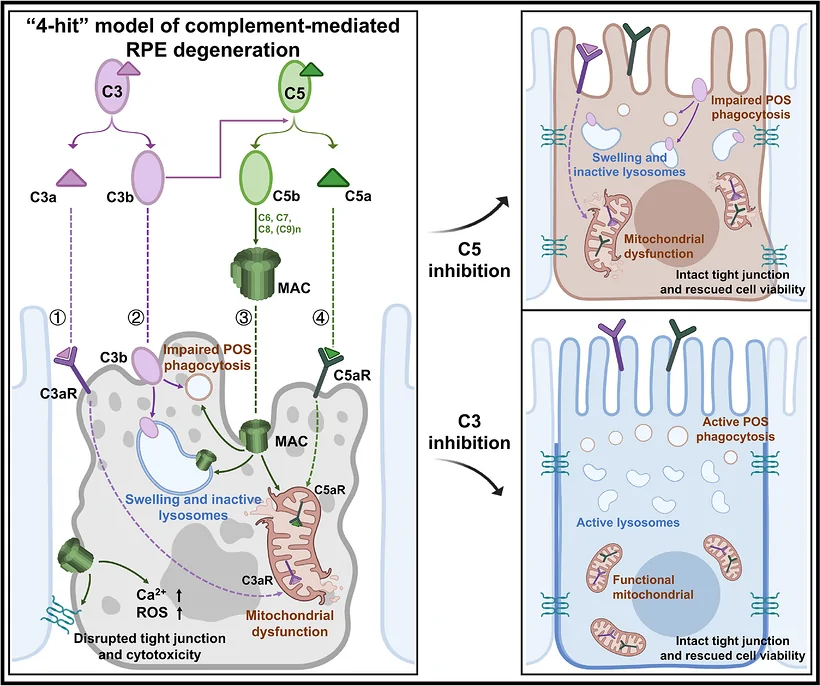
Mechanistic dissection of complement‐mediated RPE degeneration and the comprehensive protection of upstream C3 inhibition. (Top left) Complement cascade activation leads to the generation of C3a, C3b, C5a, and MAC. (Bottom left) In the absence of inhibition, these effectors contribute to a “4‐hit” pattern of degeneration in A2E‐laden RPE cells: MAC drives Ca^2+^ influx, increased ROS levels, tight‐junction disruption, and cytotoxicity; C3a and C5a signaling via C3aR and C5aR, respectively, and together with MAC, contribute to mitochondrial dysfunction; and C3b, together with MAC, contributes to lysosomal dysfunction and impaired POS phagocytosis. (Right) Comparative outcomes of inhibition strategies: C5 inhibition (top) blocks MAC formation and C5a release, preventing lytic injury. However, it fails to address the upstream formation of C3a and C3b, resulting in persistent mitochondrial dysfunction, lysosomal dysfunction, and impaired POS phagocytic capacity. C3 inhibition (bottom) intercepts the cascade upstream, preventing the generation of C3a, C3b, C5a, and MAC. This strategy provides broader rescue of organelle function and cellular homeostasis, highlighting the mechanistic advantage of upstream C3 inhibition. Created in BioRender. Chen, Z. (2026) https://BioRender.com/83rsu8o.

Our results confirm that the terminal complement pathway drives acute, MAC‐dependent RPE injury, characterized by Ca^2+^ influx, oxidative stress, cytotoxicity, and disrupted barrier function (Figure 1). While C5 blockade completely abrogates this lytic assault, consistent with the therapeutic rationale for terminal inhibitors in GA [40], our data reveal a critical distinction: preventing cell death does not preserve cell health. Under C5 inhibition, RPE cells exhibit persistent mitochondrial and lysosomal dysfunction that culminates in failure of essential phagocytic function (Figure 2). This finding raises the possibility that surviving RPE in treated patients may remain functionally compromised, potentially explaining the limited functional benefit observed in clinical trials of terminal complement inhibitors [5].

The persistence of this dysfunction points to C5‐independent pathogenic mechanisms driven by upstream effectors. We identify two such pathways. First, the anaphylatoxin C3a acts as a key mitochondrial function disruptor, as supported by the mitochondrial dysfunction induced by direct C3a stimulation and the rescue effect achieved by C3aR antagonism (Figure 3 and Figure S6). Second, activation‐generated C3b contributes to the phagolysosomal dysfunction (Figure 4). Complement dysregulation leads to excessive C3b deposition on RPE cells, and C3b signals were detected within LAMP2‐positive intracellular compartments (Figure 4G). The presence of C3b in these compartments, together with C5b9 detected in LAMP2‐positive compartments (Figure 4J), may reflect an additional burden on the lysosomal system and contribute to phagolysosomal dysfunction (Figure 4E,F). This dissection provides a mechanistic rationale for upstream C3 inhibition. By preventing C3 cleavage, this strategy blocks the generation of pathogenic complement effectors (Figure S9). C3 inhibition not only abrogated MAC‐dependent lytic injury (Figure 5A–D) but, critically, also prevented the C5‐independent pathologies, including mitochondrial dysfunction (Figure 5E–H), lysosomal dysfunction, and impaired POS phagocytosis (Figure 5K,L).

This detailed mechanistic analysis, which points to the superiority of C3 blockade, however, raises a critical question: if C3 inhibition provides broader protection, why have C3 and C5 inhibitors demonstrated broadly similar efficacy in clinical trials for GA? [4, 5]. We propose that this clinical‐mechanistic discordance can be explained by the limitations of current clinical endpoints and the significant challenges to inhibiting C3. First, the anatomical endpoint of GA lesion growth is strongly influenced by irreversible complement‐associated cell and tissue loss, including terminal pathway‐mediated injury, which both C3 and C5 inhibitors can suppress. The distinct benefits of C3 inhibition, including preservation of mitochondrial function and lysosomal degradative capacity, might be better captured by functional measures such as retinal sensitivity or visual function in surviving tissue, endpoints that are currently difficult to quantify in late‐stage GA patients. Second, complete inhibition of C3 requires a significantly higher drug concentration, and a small amount of escaped C3 will activate downstream cascade reactions through self‐amplification. This high concentration demand is rooted in two principles of complement biology: (1) a profound stoichiometric burden exists, as the physiological concentration of C3 is an order of magnitude higher than that of C5, both in serum (75–175 vs. 7–26 mg/dL) [41] and within the intraocular environment (566.90 vs. 41.05 ng/L) [42, 43]; (2) C3b, the C3 cleavage product, is the engine of the alternative pathway's powerful amplification loop and also serves as a key component of C5 convertases, which can lead to an exponential and rapid amplification of the initial signal [44]. C3, therefore, requires high‐level and sustained suppression to inhibit its large standing pool and the potent feedback mechanism. C5, in contrast, is a downstream substrate with low serum concentration and no amplification loop, and a relatively low concentration of C5 inhibitor can achieve effective terminal pathway suppression. Consequently, current intravitreal C3 regimens may fail to achieve sustained, near‐complete suppression required to manifest full mechanistic superiority. The mechanistic differentiation in the clinic may depend on achieving suprathreshold inhibition of C3. Therefore, the defining translational challenge for next‐generation C3 therapeutics lies in maintaining uninterrupted suppression of the large C3 pool and amplification loop. This imposes a drug development requirement for the long‐term maintenance of effective drug concentrations within the constraints of intravitreal dosing regimens [45, 46].

In addition to elucidating the pathogenic mechanism, our work has significant implications for the preclinical evaluation of GA therapeutics. Historically, the potency of complement inhibitors for GA has been predominantly assessed via hemolysis assays. While this gold standard effectively measures the blockade of MAC‐mediated lysis, it inherently fails to capture the complex cellular responses of nucleated RPE cells, which possess intrinsic resistance mechanisms to complement attack. As demonstrated in our study, the complete inhibition of hemolysis by C5 blockade does not translate to the full preservation of RPE cellular homeostasis. Consequently, relying solely on hemolysis assays may overestimate the therapeutic potential of candidates that block the terminal pathway but leave upstream stress signals unchecked. Therefore, utilizing physiologically relevant RPE models to evaluate functional endpoints beyond simple lysis prevention provides a more comprehensive platform for understanding drug mechanisms. In addition, it is worth noting that while this RPE monoculture system provides valuable mechanistic insights, it inherently lacks the complex multicellular interactions, such as interplay with the choriocapillaris and resident immune cells, and chronic disease progression such as drusen accumulation and progressive tissue remodeling [47, 48]. The conclusions drawn here therefore warrant further validation in more complex preclinical models in the future.

Importantly, complement activation does not operate in isolation but interacts with broader pathogenic networks in AMD. Complement activation and oxidative stress can mutually reinforce one another: in our model, complement activation increased intracellular ROS, whereas previous studies have shown that oxidative stress can impair complement regulation on RPE cells and facilitate complement activation, thereby increasing susceptibility to complement‐mediated injury [10, 11, 49]. Complement signaling is also interconnected with vascular endothelial growth factor (VEGF) signaling. C3a, C5a, and sublytic MAC have been reported to promote VEGF expression or secretion [17, 50], whereas VEGF signaling can in turn support local complement regulation by maintaining the expression of complement regulatory proteins [51]. These reciprocal interactions indicate that complement is embedded within broader oxidative and angiogenic networks in AMD. Furthermore, our observation of baseline injury in A2E‐laden RPE cells in the absence of complement activation suggests a potential ceiling for complement monotherapy. Such complement‐independent pathology cannot be addressed by complement inhibition alone and may partly contribute to the limited functional benefit observed with current C3 and C5 inhibitors [6]. Therefore, achieving a more profound therapeutic effect may require a multi‐pronged strategy that combines optimal complement inhibition with therapies targeting other fundamental disease mechanisms [6, 52]. Promising future strategies could include targeting cell death pathways such as Fas signaling [53], improving mitochondrial bioenergetics [54], or mitigating other key stressors, including oxidative damage and toxic visual‐cycle byproducts [55].

## 4 Conclusion

In summary, this study provides a mechanistic rationale for upstream C3 inhibition in complement‐mediated RPE degeneration relevant to atrophic AMD and GA. Our findings demonstrate that the complement‐mediated RPE degeneration extends beyond MAC‐dependent lysis and is substantially driven by C5‐independent damage to mitochondrial and phagolysosomal systems associated with C3 activation products. Consequently, upstream C3 inhibition provided broader protection across the measured RPE phenotypes than terminal C5 blockade. These findings support further evaluation of sustained and effective C3 inhibition as a therapeutic strategy for GA secondary to AMD.

## 5 Materials and Methods

### 5.1 Reagents

The complement inhibitors used in this study are summarized in Table 1. Other reagents include: DMEM/F12 (1:1; Gibco, cat#11320033), fetal bovine serum (Gibco, cat#10099158), penicillin/streptomycin (Gibco, cat#15140122), NHS (Gemini, cat#100‐512), SMM 293‐TII medium (Sinobiological, cat#M293TII), polyethylenimine (PEI) (Biohub International Trade, cat#78PEI4000), Ni‐NTA agarose (Smart Life Sciences, cat#SA004025), Avacincaptad pegol (synthesized by MCE), A2E (MCE, cat#HY‐134928A), C5b6 (CompTech, cat#122), C8 (CompTech, cat#125), C3a (MCE, cat#HY‐P7862), C5a (MCE, cat#HY‐P7864), C3aR antagonist (MCE, cat#HY‐101502A), C5aR antagonist (MCE, cat#HY‐17627), CD59‐Fc fusion protein (MCE, cat#HY‐P700683), TrypLE Express (Gibco, cat#12604013), anti‐C5b9 (Abcam, cat#ab66768, clone aE11), anti‐C3/C3b/iC3b/C3d (Biolegend, cat#846302, clone 1H8), goat anti‐mouse 647 secondary antibody (Abcam, cat#ab150115), goat anti‐rabbit 488 secondary antibody (Abcam, cat#ab150077), PBS (Gibco, cat#10010023), DAPI (Beyotime, cat#C1002), 4‐Chamber Glass Bottom Dish (Cellvis, cat#D35C4‐20‐1.5‐N), formaldehyde (Sangon Biotech, cat#E672002), Triton‐X 100 (Solarbio, cat#T8200), anti‐LAMP2 (Abcam, cat#ab199947, clone EPR19531), mounting media containing DAPI (Servicebio, cat#G1407), Fluo‐4 AM (Yeasen, cat#40704ES50), DCFH‐DA (Beyotime, cat#S0033S), MitoNeoD (Beyotime, cat#S0062S), LDH Cytotoxicity Assay Kit (Beyotime, cat#C0017), CCK8 (Solarbio, cat#CA1210), CellTiter‐Lumi Steady Plus II (Beyotime, cat#C0059S), 12‐well Transwell inserts (Corning, cat#3460), laminin‐521 (Biolaminin, cat#LN521‐02), anti‐ZO1 (Invitrogen, cat#33‐9100, clone ZO1‐1A12), goat anti‐mouse 488 secondary antibody (Abcam, cat#ab150113), anti‐p‐RIP3 (Abcam, cat#Ab209384, clone EPR9627), anti‐Active Caspase‐3 (Immunoway, cat#YM3431, clone 5E1), goat anti‐rabbit 647 secondary antibody (Abcam, cat#ab150083), TMRE (Beyotime, cat#C2001S), MitoTracker Green FM (ThermoFisher, cat#A66441), DilC1(5) (MCE, cat#HY‐D1075), Hoechst (Beyotime, cat#C1029), anti‐TOMM20 (Proteintech, cat#11802‐1‐AP, RRID: AB_2207530), anti‐Cytochrome c (Beyotime, cat#AC908, clone 6H2.B4), anti‐LAMP2 (Abcam, cat#ab25631, clone H4B4), anti‐Cathepsin D (Proteintech, cat#21327‐1‐AP, RRID: AB_10733646), LysoTracker Green DND‐26 (ThermoFisher, cat#A66438), pHrodo (ThermoFisher, cat#P35370), bovine photoreceptor outer segments (POS) (InVision Bioresources, cat#98740), C5a ELISA kit (Quidel, cat#A021), APC anti‐CD46 (Biolegend, cat#352405, clone TRA‐2‐10), APC anti‐CD55 (Biolegend, cat#311311, clone JS11), APC anti‐CD59 (Biolegend, cat#304711, clone p282 (H19)), Cyanine 5 monosuccinimidyl ester (AAT Bioquest, cat#151), bovine serum albumin (BSA) (Beyotime, cat#ST2254).

**TABLE 1:** Summary of complement inhibitors used in this study.

Inhibitor	Target	Identity	Mechanism of action	Source
C3‐Nf	C3	Nanofitin (8.4 kDa)	Prevents C3 cleavage by C3 convertases	Recombinant expression
C5‐DNb	C5	Dual‐epitope nanobody (42.0 kDa)	Binds C5 at two distinct epitopes, completely preventing its conformational transformation and cleavage	Recombinant expression
C3aR Antagonist	C3aR	Small molecule	Selectively antagonizes the C3aR	MCE, cat#HY‐101502A
C5aR Antagonist	C5aR	Small molecule	Selectively antagonizes the C5aR	MCE, cat#HY‐17627
CD59‐Fc fusion protein	C5b8/9	Fusion protein (35.7 kDa)	Prevents MAC pore formation	MCE, cat#HY‐P700683

### 5.2 Cell Culture

The human RPE cell line ARPE‐19 [56] (ATCC number CRL‐2302, purchased from Procell) was cultured in DMEM/F12 supplemented with 10% fetal bovine serum and 100 U/mL penicillin/streptomycin at 37°C and 5% CO_2_. The cell line was authenticated by STR profiling by the supplier, and the cells were routinely tested for mycoplasma contamination and were found to be negative. Cells were passaged upon reaching 80%–90% confluence. For experiments, cells were grown on 12‐, 24‐, or 96‐well plates or glass slides for 3–5 days until the cells reached confluence. After confluence, fetal bovine serum was removed completely for the final 1–2 days (1 media change) before A2E and NHS/HI‐NHS treatments and subsequent measurements. In all experiments, 40% NHS and 40% HI‐NHS (56°C, 30 min) were used as the positive and negative controls for complement activation, respectively.

### 5.3 Recombinant Expression and Purification

C3‐Nf, C5‐DNb, C5‐SNb1, C5‐SNb2, human C7, and C9 were cloned into the pCDNA3.1(+) plasmid with the addition of the linker (GGGGS) and 6xHis (HHHHHH) at the C‐terminus of the protein. The sequences of all plasmids were verified by sequencing before expression. HEK293‐F cells were cultured in SMM 293‐TII medium at 5% CO_2_ and 37°C for 4–5 days before expression. The cells were then transfected with the plasmid and PEI at a final concentration of 1 and 3 µg/mL, respectively, at a density of 1.5‐2.0 × 10^6^ cells/mL. Expressed proteins in the supernatant were purified by Ni‐NTA agarose. For purification by Ni‐NTA agarose, 3 mL Ni‐NTA agarose was added to 1 L of supernatant from the cell culture medium and incubated at 4°C overnight. The Ni‐NTA agarose was recovered through Gravity Chromatography Columns and washed with 10 mM imidazole in PBS. After washing, the protein was eluted from the Ni‐NTA agarose with 200 mM imidazole in PBS and concentrated. Molecular weight and purity of expressed proteins were confirmed by SDS‐PAGE (Figure S10).

### 5.4 C3b and C5b‐9 Deposition and Lysosomal Colocalization Analysis

Cells (1 × 10^5^) were grown on 12‐well plates and then incubated in DMEM/F12 for 1 day after confluence. Cells were then incubated with 450 µL, 30 µM A2E for 24 h before washing, and subsequently incubated with 450 µL of HI‐NHS and NHS, with or without complement inhibitor, for another 24 h. After subsequent treatment with A2E and HI‐NHS/NHS, the cells were harvested with TrypLE Express and resuspended in PBS and 1% BSA for 30 min. Anti‐C5b9 (1:200) or anti‐C3b (1:200) was applied for 30 min at 4°C. The cells were washed three times and incubated with a goat anti‐mouse 647 secondary antibody (1:1000) for 30 min at 4°C in the dark.

To assess the localization of C3b‐ and C5b9‐associated signals within lysosomal compartments, colocalization analysis with the lysosomal marker LAMP2 was performed by confocal immunofluorescence microscopy. Cells (5 × 10^4^) were plated on a 4‐Chamber Glass Bottom Dish and grown for 3–5 days before being incubated in DMEM/F12 for 1 day. Cells were then incubated with 300 µL, 30 µM A2E for 24 h before washing, and subsequently incubated with 300 µL HI‐NHS and NHS with or without C3‐Nf (40 µM) or C5‐DNb (20 µM) for 2 h. After subsequent treatment with A2E and HI‐NHS/NHS, cells were washed and fixed with 4% formaldehyde for 10 min at room temperature (RT) and then permeabilized with 0.3% Triton X‐100 for 10 min at RT. Nonspecific binding was blocked by treatment of the cells with 3% BSA, followed by incubation with anti‐LAMP2 (1:200) and anti‐C5b9 (1:200) or anti‐C3b (1:200) for 16 h at 4°C. The cells were washed and then incubated with goat anti‐mouse 647 secondary antibody (1:1000) and goat anti‐rabbit 488 secondary antibody (1:1000) and washed as described above. The cells were coated with mounting media containing DAPI, covered, and analyzed by Leica STELLARIS 8 Falcon (Leica Microsystems GmbH, Wetzlar, Germany).

### 5.5 C3a and C5a Release Detection

The culture supernatants from the RPE cell experiment or hemolysis experiment were collected for the detection of C3a and C5a contents. The C3a content was determined as described in the previous study [39]. The C5a content was determined by the C5a ELISA kit according to the manufacturer's recommendations.

### 5.6 Cytosolic Calcium Measurement

The calcium‐sensitive dye Fluo‐4 AM (2 mM) was used to measure the cytosolic calcium level. Cells (1 × 10^5^) were grown on 12‐well plates and then incubated in DMEM/F12 for 1 day after confluence. Cells were then incubated with 450 µL, 30 µM A2E for 24 h before washing, and subsequently incubated with 450 µL HI‐NHS with or without complement component for another 24 h: C3a (2 µg/mL) and C5a (2 µg/mL), or NHS with or without complement inhibitors: C3aR antagonist (10 µM), C5aR antagonist (10 µM), C3‐Nf (40 µM), C5‐DNb (20 µM), and CD59‐Fc fusion protein (40 µM). After subsequent treatment with A2E and HI‐NHS/NHS, the cells were harvested by TrypLE Express, pelleted, and resuspended in 4 µM Fluo‐4 AM at 37°C for 1 h. After staining, cells were then washed and resuspended in 200 µL of PBS containing 250 ng/mL DAPI and run on a FACSCalibur machine (BD Biosciences) and analyzed by FlowJo (version 7.6.5 software; Tree Star, Ashland, OR).

### 5.7 Reactive Oxygen Species (ROS) Measurement

The DCFH‐DA (1:1000) was used to measure ROS levels. Cells (1 × 10^5^) were grown on 12‐well plates and then incubated in DMEM/F12 for 1 day after confluence. Cells were then incubated with 450 µL, 30 µM A2E for 24 h before washing, and subsequently incubated with 450 µL HI‐NHS with or without complement component for another 24 h: C3a (2 µg/mL) and C5a (2 µg/mL), or NHS with or without complement inhibitors: C3aR antagonist (10 µM), C5aR antagonist (10 µM), C3‐Nf (40 µM), C5‐DNb (20 µM), and CD59‐Fc fusion protein (40 µM). After subsequent treatment of A2E and HI‐NHS/NHS, the cells were harvested by TrypLE Express, pelleted, and resuspended in DCFH‐DA (1:1000) at 37°C for 1 h. After staining, cells were then washed and resuspended in 200 µL of PBS containing 250 ng/mL DAPI and run on a FACSCalibur machine (BD Biosciences) and analyzed by FlowJo (version 7.6.5 software; Tree Star, Ashland, OR).

### 5.8 CCK‐8 Assay

Cells (1 × 10^4^) were grown on 96‐well plates and then incubated in DMEM/F12 for 1 day after confluence. Cells were then incubated with 100 µL, 30 µM A2E for 24 h before washing, and subsequently incubated with 100 µL HI‐NHS and NHS with or without complement component for another 24 h: C3a and C5a or complement inhibitors: C3aR antagonist (10 µM), C5aR antagonist (10 µM), C3‐Nf (40 µM), C5‐DNb (20 µM), and CD59‐Fc fusion protein (40 µM).

For the CCK‐8 assay, 100 µL CCK8 (1:10) was directly added to cells after supernatant collection. The cells were incubated at 37°C for 1 h in the CCK‐8 assay. The viability of the cells was then evaluated by measuring the absorbance at 450 nm on a microplate reader (SpectraMax Gemini XPS/EM Microplate Readers, Molecular Devices, USA).

### 5.9 Trans‐Epithelial Electrical Resistance (TEER) Measurement and Tight Junction Detection

Cells (2 × 10^5^ cells per well) were plated on 12‐well Transwell inserts with polycarbonate membranes pre‐coated with laminin‐521. During the first week, the culture medium with 10 mM nicotinamide supplementation was replaced every other day, followed by daily changes starting from the second week until day 28. TEER values were recorded starting on Day 7 using a Millicell ERS‐2 Voltohmmeter (Millipore, Billerica, MA) to evaluate the integrity and maturation of the cell monolayer. The value for cell monolayers was determined by subtracting the TEER for filters without cells and then multiplying by the surface area of the filters. Cell monolayers were considered stable when TEER was repeatedly measured as ∼40–60 Ω*cm^2^ [49].

After 28 days of culture on 12‐well Transwell inserts, the cells were then incubated with 250 µL, 80 µM A2E for 24 h before washing, and subsequently incubated with 250 µL HI‐NHS and NHS with or without complement inhibitors: C5aR antagonist (10 µM), C3‐Nf (40 µM), C5‐DNb (20 µM), and CD59‐Fc fusion protein (40 µM) for another 48 h. TEER values were recorded every 24 h during treatments.

For tight junction detection, cells on 12‐well Transwell inserts after treatment and washing were fixed with ice‐cold methanol and permeabilized with 0.1% Triton X‐100. Nonspecific binding was blocked by treatment of the cells with 3% BSA, followed by incubation with anti‐ZO1 (1:200) for 16 h at 4°C. The cells were washed and then incubated with goat anti‐mouse 488 secondary antibody (1:1000) and washed as described above. The cells were coated with mounting media containing DAPI, covered, and analyzed by Leica STELLARIS 8 Falcon (Leica Microsystems GmbH, Wetzlar, Germany).

### 5.10 Mitochondrial Function Detection

#### 5.10.1 Mitochondrial Superoxide Measurement

The MitoNeoD (1:1000) was used to measure mitochondrial superoxide level. Cells (1 × 10^5^) were grown on 12‐well plates and then incubated in DMEM/F12 for 1 day after confluence. Cells were then incubated with 450 µL of 30 µM A2E for 24 h. The cells were then subjected to 24 h treatment with following conditions: 450 µL HI‐NHS with or without complement C3a (2 µg/mL) and C5a (2 µg/mL), or 450 µL NHS with or without complement inhibitors: C3aR antagonist (10 µM), C5aR antagonist (10 µM), C3‐Nf (40 µM), C5‐DNb (20 µM), and CD59‐Fc fusion protein (40 µM). Following these treatments, cells were harvested by TrypLE Express, pelleted, and resuspended in MitoNeoD (1:1000) at 4°C for 1 h. After staining, cells were then washed and resuspended in 200 µL of PBS containing 250 ng/mL DAPI and run on a FACSCalibur machine (BD Biosciences), and analyzed by FlowJo (version 7.6.5 software; Tree Star, Ashland, OR).

#### 5.10.2 Mitochondrial Membrane Potential Measurement

TMRE (1:1000) was used to measure mitochondrial membrane potential. Cells (1 × 10^5^) were grown on 12‐well plates and then incubated in DMEM/F12 for 1 day after confluence. Cells were then incubated with 450 µL of 30 µM A2E for 24 h. The cells were then subjected to 24 h treatment with the following conditions: 450 µL HI‐NHS with or without complement C3a (2 µg/mL) and C5a (2 µg/mL), or 450 µL NHS with or without complement inhibitors: C3aR antagonist (10 µM), C5aR antagonist (10 µM), C3‐Nf (40 µM), C5‐DNb (20 µM), and CD59‐Fc fusion protein (40 µM). Following these treatments, cells were harvested by TrypLE Express, pelleted, and resuspended in TMRE (1:1000) at 37°C for 1 h. After staining, cells were then washed and resuspended in 200 µL of PBS containing 250 ng/mL DAPI and run on a FACSCalibur machine (BD Biosciences) and analyzed by FlowJo (version 7.6.5 software; Tree Star, Ashland, OR).

The TMRE was also detected by immunofluorescence microscopy. Cells (5 × 10^4^) were plated on a 4‐Chamber Glass Bottom Dish and grown for 3–5 days before being incubated in DMEM/F12 for 1 day. Cells were then incubated with 300 µL, 30 µM A2E for 24 h. After washing, the cells were subjected to 24 h treatment with HI‐NHS or NHS. After washing with PBS, cells were stained with TMRE (1:1000) at 37°C for 1 h. The dishes were washed three times in PBS and incubated with Hoechst (1:100) at 37 °C for 30 min, and washed as described above. The cells were analyzed by Leica STELLARIS 8 Falcon (Leica Microsystems GmbH, Wetzlar, Germany).

#### 5.10.3 TOMM20 Detection

Cells (5 × 10^4^) were plated on a 4‐Chamber Glass Bottom Dish and grown for 3–5 days before being incubated in DMEM/F12 for 1 day. Cells were then incubated with 300 µL of 30 µM A2E for 24 h. After washing, the cells were subjected to 24 h treatment with HI‐NHS or NHS. Cells were washed and fixed with 4% formaldehyde for 10 min at RT and then permeabilized with 0.1% Triton X‐100 for 10 min at RT. Nonspecific binding was blocked by treatment of the cells with 3% BSA, followed by incubation with anti‐TOMM20 (1:200) for 16 h at 4°C. The cells were washed and then incubated with goat anti‐rabbit 488 secondary antibody (1:1000). The cells were coated with mounting media containing DAPI, covered, and analyzed by Leica STELLARIS 8 Falcon (Leica Microsystems GmbH, Wetzlar, Germany).

### 5.11 Mitochondrial Mass Measurement

The MitoTracker Green FM (250 nM) was used to measure mitochondrial mass. Cells (1 × 10^5^) were grown on 12‐well plates and then incubated in DMEM/F12 for 1 day after confluence. Cells were then incubated with 450 µL of 30 µM A2E for 24 h. After washing, the cells were subjected to 24 h treatment with the following conditions: 450 µL HI‐NHS with or without complement C3a (2 µg/mL) and C5a (2 µg/mL), or 450 µL NHS with or without complement inhibitors: C3aR antagonist (10 µM), C5aR antagonist (10 µM), C3‐Nf (40 µM), C5‐DNb (20 µM), and CD59‐Fc fusion protein (40 µM). Following these treatments, cells were harvested by TrypLE Express, pelleted, and resuspended in MitoTracker Green FM (250 nM) at 37°C for 1 h. After staining, cells were then washed and resuspended in 200 µL of PBS containing 250 ng/mL DAPI and run on a FACSCalibur machine (BD Biosciences), and analyzed by FlowJo (version 7.6.5 software; Tree Star, Ashland, OR).

### 5.12 Cytochrome c Release Detection

Cells (5 × 10^4^) were plated on a 4‐Chamber Glass Bottom Dish and grown for 3–5 days before being incubated in DMEM/F12 for 1 day. Cells were then incubated with 300 µL of 30 µM A2E for 24 h. After washing, the cells were subjected to 24 h treatment with the following conditions: 300 µL HI‐NHS, or 300 µL NHS with or without complement inhibitors: C3aR antagonist (10 µM), C3‐Nf (40 µM), and C5‐DNb (20 µM). Following these treatments, cells were washed and fixed with 4% formaldehyde for 10 min at RT and then permeabilized with 0.3% Triton X‐100 for 10 min at RT. Nonspecific binding was blocked by treatment of the cells with 3% BSA, followed by incubation with anti‐cytochrome c (1:200) for 16 h at 4°C. The cells were washed and then incubated with goat anti‐mouse 647 secondary antibody (1:1000) and washed as described above. The cells were coated with mounting media containing DAPI, covered, and analyzed by Leica STELLARIS 8 Falcon (Leica Microsystems GmbH, Wetzlar, Germany).

### 5.13 Lysosomal Function Detection

#### 5.13.1 LAMP2 Detection

Cells on 12‐well Transwell inserts after treatment and washing were fixed with ice‐cold methanol and permeabilized with 0.1% Triton X‐100. Nonspecific binding was blocked by treatment of the cells with 3% BSA, followed by incubation with anti‐LAMP2 (1:200) for 16 h at 4°C. The cells were washed and then incubated with goat anti‐rabbit 488 secondary antibody (1:1000) and washed as described above. The cells were coated with mounting media containing DAPI, covered, and analyzed by Leica STELLARIS 8 Falcon (Leica Microsystems GmbH, Wetzlar, Germany).

#### 5.13.2 Cathepsin D Puncta Detection

Cells (5 × 10^4^) were plated on a 4‐Chamber Glass Bottom Dish and grown for 3–5 days before being incubated in DMEM/F12 for 1 day. Cells were then incubated with 300 µL of 30 µM A2E for 24 h before washing, and subsequently incubated with 300 µL of HI‐NHS and NHS for 24 h. After subsequent treatment with A2E and HI‐NHS/NHS, cells were washed and fixed with 4% formaldehyde for 10 min at RT and then permeabilized with 0.3% Triton X‐100 for 10 min at RT. Nonspecific binding was blocked by treatment of the cells with 3% BSA, followed by incubation with anti‐Cathepsin D (1:200) for 16 h at 4°C. The cells were washed and then incubated with goat anti‐rabbit 488 secondary antibody (1:1000) and washed as described above. The cells were coated with mounting media containing DAPI, covered, and analyzed by Leica STELLARIS 8 Falcon (Leica Microsystems GmbH, Wetzlar, Germany).

#### 5.13.3 LysoTracker Puncta Detection

Cells (5 × 10^4^) were plated on a 4‐Chamber Glass Bottom Dish (Cellvis) and grown for 3–5 days before being incubated in DMEM/F12 for 1 day. Cells were then incubated with 300 µL, 30 µM A2E for 24 h before washing, and subsequently incubated with 300 µL HI‐NHS and NHS with or without complement component for another 24 h: C3a (2 µg/mL) and C5a (2 µg/mL), or complement inhibitors: C3aR antagonist (10 µM), C5aR antagonist (10 µM), C3‐Nf (40 µM), C5‐DNb (20 µM), and CD59‐Fc fusion protein (40 µM). After washing with PBS, cells were stained with LysoTracker Green DND‐26 (250 nM) at 37°C for 1 h. The dishes were washed three times in PBS and incubated with Hoechst (1:100) at 37°C for 30 min, and washed as described above. The cells were analyzed by Leica STELLARIS 8 Falcon (Leica Microsystems GmbH, Wetzlar, Germany).

### 5.14 POS Phagocytosis Detection

Cells (1 × 10^5^) were grown on 12‐well plates and then incubated in DMEM/F12 for 1 day after confluence. Cells were then incubated with 450 µL, 30 µM A2E for 24 h before washing, and subsequently incubated with 450 µL HI‐NHS and NHS with or without complement component for another 24 h: C3a (2 µg/mL) and C5a (2 µg/mL), or complement inhibitors: C3aR antagonist (10 µM), C5aR antagonist (10 µM), C3‐Nf (40 µM), C5‐DNb (2.5, 10, 20, and 40 µM), and CD59‐Fc fusion protein (40 µM). After subsequent treatment with A2E and HI‐NHS/NHS, the cells were fed with pHrodo^−^labelled bovine photoreceptor outer segments (POS) at 10 POS/RPE cell at 37°C for 5 h. Untreated cells were used as a control. After 5 h, cells were harvested by TrypLE Express, pelleted, washed, and resuspended in 200 µl of PBS containing 250 ng/mL DAPI and run on a FACSCalibur machine (BD Biosciences), and analyzed by FlowJo (version 7.6.5 software; Tree Star, Ashland, OR).

The POS phagocytic function was also detected by immunofluorescence microscopy. Cells (5 × 10^4^) were plated on a 4‐Chamber Glass Bottom Dish and grown for 3–5 days before being incubated in DMEM/F12 for 1 day. Cells were then incubated with 300 µL of 30 µM A2E for 24 h before washing, and subsequently incubated with 300 µL HI‐NHS and NHS for another 24 h. After subsequent treatment with A2E and HI‐NHS/NHS, the cells were fed with pHrodo^−^labelled POS 10 POS/RPE cells at 37°C for 5 h. After 5 h, the cells were washed and analyzed by Leica STELLARIS 8 Falcon (Leica Microsystems GmbH, Wetzlar, Germany).

### 5.15 Statistical Analysis

Data were analyzed using GraphPad Prism 9. Comparisons between two groups were performed using an unpaired t‐test. Comparisons among multiple groups were performed using one‐way ANOVA with Tukey's multiple comparison test. Data are presented as mean ± SEM.

## Author Contributions

Z.C.: conceptualization, methodology, data curation, investigation, formal analysis, visualization, writing – original draft, writing – review & editing. M.W.: methodology, investigation, writing – review & editing. H.L.: conceptualization, methodology, formal analysis, writing – original draft, writing – review & editing. F.Q.: conceptualization, formal analysis, supervision, funding acquisition, project administration, resources, writing – review & editing.

## Funding

This work was supported by grants from the Beijing Frontier Research Center for Biological Structure (Grant No. 041500002) and the National Natural Science Foundation of China (Project No. 82473854).

## Conflicts of Interest

The authors declare no conflicts of interest.

## Supporting information




**Supporting File**: advs78539‐sup‐0001‐SuppMat.docx.

## Data Availability

The data that support the findings of this study are available from the corresponding author upon reasonable request.
