# Supplementary material for: A Mechanistic Dissection of Multifaceted Complement Attack in Age‐Related Macular Degeneration Reveals the Superiority of C3 Inhibition
Source: Adv Sci (Weinh). 2026 Oct 28:e78539. Online ahead of print. doi: 10.1002/advs.78539 (PMC13715677; doi:10.1002/advs.78539)
Supplement: Supplementary file 1 [file ADVS-9999-e78539-s001.docx]

**Supporting Information
A Mechanistic Dissection of Multifaceted Complement Attack in Age-Related Macular Degeneration Reveals the Superiority of C3 Inhibition**

Zhidong Chen^1^, Mingshuang Wang^2^, Huiqin Liu^1*^, Feng Qian^1*^

1 School of Pharmaceutical Sciences, Beijing Visual Science and Translational Eye Research Institute (BERI), Tsinghua Medicine, and Beijing Frontier Research Center for Biological Structure, Tsinghua University, Beijing, P. R. China

2 Quaerite Biopharm Research, Beijing 100195, P.R. China

* **For correspondence**:

Huiqin Liu, huiqin-liu@mail.tsinghua.edu.cn; Feng Qian, qianfeng@tsinghua.edu.cn

**SI** **Materials and Methods**

**Hemolysis assays**

The classical pathway (CP) and alternative pathway (AP)-mediated hemolysis assays were performed as described in the previous study [1].

**A2E Loading and visualization**

Cells (5×10^4^) were plated on a 4-Chamber Glass Bottom Dish and grown for 3-5 days before being incubated in DMEM/F12 for 1 day. Cells were then incubated with 300 µl, 30 µM A2E for 24h. The cells were then washed and analyzed by Leica STELLARIS 8 Falcon (Leica Microsystems GmbH, Wetzlar, Germany).

**Detection of surface expression level of CD46, CD55, and CD59**

Cells (1×10^5^) were grown on 12-well plates and then incubated in DMEM/F12 for 1 day after confluence. Cells were then incubated with 450 µl, 30 µM A2E for 24 h. After A2E treatment, the cells were harvested by TrypLE^TM^ Express, pelleted, and resuspended in APC anti-CD46 (1:100), or APC anti-CD55 (1:100), or APC anti-CD59 (1:100). After staining, cells were then washed and resuspended in 200 μl of PBS containing 250 ng/ml DAPI and run on a FACSCalibur machine (BD Biosciences) and analyzed by FlowJo (version 7.6.5 software; Tree Star, Ashland, OR).

**Cytotoxicity assays: LDH release and CellTiter-Lumi**

Cells (1×10^4^) were grown on 96-well plates and then incubated in DMEM/F12 for 1 day after confluence. Cells were then incubated with 100 µl, 30 µM A2E or DMEM/F12 for 24 h before washing and subsequently incubated with 100 µl HI-NHS and NHS.

After treatment, the supernatants were collected for LDH release detection. The LDH Cytotoxicity Assay Kit was used to quantify LDH released from cultured RPE cells into the culture medium according to the manufacturer’s recommendations. Briefly, a reaction mixture consisting of was prepared, and 40 μL was added directly to 80 μL of the cell supernatant. After incubation at 25 °C for 30 minutes, absorbance was read using a spectrophotometer at 490 nm.

For CellTiter-Lumi assays, 100 µl CellTiter-Lumi^TM^ Steady Plus II was directly added to cells after supernatant collection, respectively. The cells were incubated at 25 °C for 10 minutes in the CellTiter-Lumi assay. The viability of the cells was then detected by PerkinElmer EnVision I.

**Apoptosis and necrosis marker detection**

Apoptosis marker (Active-caspase 3) and necrosis marker (Phospho-RIP3) were detected by immunofluorescence microscopy. Cells (5×10^4^) were plated on a 4-Chamber Glass Bottom Dish and grown for 3-5 days before being incubated in DMEM/F12 for 1 day. Cells were then incubated with 300 µl, 30 µM A2E for 24h before washing, and subsequently incubated with 300 µl HI-NHS and NHS. After subsequent treatment of A2E and HI-NHS/NHS, cells were washed and fixed with 4% formaldehyde for 10 min at RT and then permeabilised with 0.3% Triton X-100 for 10 min at RT. Nonspecific binding was blocked by treatment of the cells with 3% BSA, followed by incubation with anti-p-RIP3 (1:200) and anti-Active Caspase-3 (1:200) for 16 h at 4 °C. The cells were washed and then co-incubated with goat anti-mouse 488 secondary antibody (1:1000) and goat anti-rabbit 647 secondary antibody (1:1000) and washed as described above. The cells were coated with mounting media containing DAPI, covered, and analyzed by Leica STELLARIS 8 Falcon (Leica Microsystems GmbH, Wetzlar, Germany).

**Purified membrane attack complex (MAC) assembly assay**

To determine the direct effects of the MAC independently of upstream complement activation, A2E-laden ARPE-19 cells were first incubated with purified human C5b6 (30 µg/mL) together with C7 (25 µg/mL) for 10 min at 37 °C. Subsequently, purified C8 (25 µg/mL) and C9 (50 µg/mL) were added. Control cells received C5b6 and C7 followed by PBS instead of C8 and C9. For experiments evaluating acute MAC-induced calcium influx, cells were preloaded with 2 µM Fluo-4 AM, TMRE, and Hoechst 33342 for 30 min at 37 °C before replacing the staining solution with phenol red-free DMEM/F12. Images were acquired immediately before addition of C8 and C9 to establish baseline fluorescence and continuously after MAC assembly under identical microscope settings. For experiments evaluating prolonged MAC injury, cells were incubated with MAC for 24 h before subsequent analyses. Mitochondrial membrane potential, mitochondrial mass, lysosomal function, POS phagocytosis, LDH release, and immunofluorescence staining for C5b9 and LAMP2 were then performed using the procedures described in the corresponding Methods sections.

**Figure S1. Characterization of complement hemolytic activity in Normal Human Serum (NHS) versus Heat-Inactivated NHS (HI-NHS)**. Assessment of CP and AP activity of NHS by hemolysis. (**A**) Sensitized sheep red blood cells (RBCs) for CP and (**B**) rabbit RBCs for AP were incubated with increasing concentration (0%-80%) of NHS or HI-NHS. The degree of hemolysis was detected by the optical density at 412 nm (OD412) in supernatant, indicative of the hemoglobin content and hemolysis degree.

**Figure S2. A2E loading sensitizes RPE cells to complement activation by reducing surface complement regulatory proteins.** (**A**) Representative confocal images of A2E internalization in ARPE-19 cells, and the fluorescence of A2E was detected by AF488 (490/525 nm), AF594 (590/618 nm), and AF647 (650/671 nm) simultaneously. (**B, C**) Surface protein level of complement regulators on ARPE-19 cells with or without A2E loading, analyzed by flow cytometry and quantified as median fluorescence intensity (MFI). (**D-F**) Quantification of complement activation products in A2E-laden cells treated with 40% NHS or HI-NHS. (**D**) C3b deposition on the cell surface, measured by flow cytometry and quantified as MFI. (**E**) C3a release into the cell culture supernatant, measured by ELISA and quantified as OD450. (**F**) C5a release into the cell culture supernatant, measured by ELISA and quantified as OD450. Data are presented as mean ± SEM from n=3 independent experiments. Statistical analyses were performed using GraphPad Prism 9 (https://www.graphpad.com) by one-way ANOVA with Tukey's multiple comparison test. ∗∗∗∗=p < 0.0001, ∗∗∗=p < 0.001, ∗∗=p < 0.01, ∗=p < 0.05, ns=non-significant.

**Figure S3. Complement activation induces cytotoxicity in A2E-laden RPE cells via mixed apoptotic and necroptotic pathways.** ARPE-19 cells, with or without A2E loading (30 µM, 24 h), were treated for 24 h with 40% NHS or 40% HI-NHS. (**A**) Cell membrane leakage was assessed by measurement of LDH release in the culture supernatant and quantified by OD490. (**B**) Cell viability was assessed by intracellular ATP level, measured using the CellTiter-Lumi assay and quantified as luminescence relative light units (RLU). (**C**) Apoptosis marker active-caspase3 level, analyzed by immunofluorescence and quantified as “MFI x total spot area / cell number”, referred as “Total signals / cell”. (**D**) Necroptosis marker phospho-RIP3 level, analyzed by immunofluorescence and quantified as “MFI x total spot area / cell number”, referred as “Total signals / cell”. (**E**) Representative confocal images of co-stained for active-caspase3 (green) and phospho-RIP3 (red). Nuclei were counterstained with DAPI (blue). Data are presented as mean ± SEM from n=3-4 independent experiments. Statistical analyses were performed using GraphPad Prism 9 (https://www.graphpad.com) by one-way ANOVA with Tukey's multiple comparison test. ∗∗∗∗=p < 0.0001, ∗∗∗=p < 0.001, ∗∗=p < 0.01, ∗=p < 0.05, ns=non-significant.

**Figure S4. The dual-epitope anti-C5 fusion protein (C5-DNb) exhibits superior efficacy in inhibiting AP-mediated hemolysis**. Dose-response curves of AP-mediated hemolysis inhibition.

**Figure S5. Purified MAC induces lytic injury in A2E-laden RPE cells**. (**A**) Quantification of C5b9 deposition following incubation of A2E-laden ARPE-19 with purified C5b6, C7, C8 and C9. Cells receiving C5b6 and C7 without C8 or C9 served as negative controls. (**B**) Cytosolic Ca^2+^ level was measured using the Fluo-4 AM probe by IF staining and quantified as quantified as “MFI x total spot area / cell number”, referred as “Total signals / cell”. (**C**) Reactive oxygen species (ROS) level was measured using the DCFH-DA probe by flow cytometry and quantified as MFI. (**D**) Cell membrane leakage was assessed by measurement of LDH release in the supernatant and quantified by OD490. Data are presented as mean ± SEM from n = 3 independent experiments. Statistical significance was determined using one-way ANOVA followed by Tukey's multiple-comparison test.

**Figure S6. The C3a and the terminal complement effectors, C5a and MAC, contribute to the mitochondrial dysfunction in A2E-laden RPE cells**. In (**A-C**), A2E-laden ARPE-19 cells were treated with 40% HI-NHS, 40% NHS, or the indicated treatments. (**A**) Mitochondrial superoxide level was measured using MitoNeoD by flow cytometry and quantified as MFI. (**B**) Mitochondrial membrane potential was measured using TMRE by flow cytometry and quantified as MFI. (**C**) Mitochondrial mass was measured using MitoTracker^TM^ Green FM by flow cytometry and quantified as MFI. Data are presented as normalized relative values calculated as: Relative Value (%) = [(Sample − Min) / (Max − Min)] × 100. In (**D-E**), A2E-laden RPE cells were treated with purified C3a, C3b, C5a, or assembled MAC under serum-free condition. (**D**) Mitochondrial membrane potential was measured using the DilC1(5) by flow cytometry and quantified as MFI. (**E**) Mitochondrial mass was measured using the MitoTracker^TM^ Green FM by flow cytometry and quantified as MFI. Data are presented as mean ± SEM from n=3 independent experiments. Statistical analyses were performed using GraphPad Prism 9 (https://www.graphpad.com) by one-way ANOVA with Tukey's multiple comparison test. ∗∗∗∗=p < 0.0001, ∗∗∗=p < 0.001, ∗∗=p < 0.01, ∗=p < 0.05, ns=non-significant.

**Figure S7. MAC induces lysosomal dysfunction in A2E-laden RPE cell.** (**A**) Acidic lysosomal compartments measured by IF staining for LysoTracker^TM^ Green DND-26, analyzed by quantification of LysoTracker-positive puncta number per cell. (**B**) POS phagocytic function assessed by pHrodo-POS signal and analyzed by flow cytometry and quantified as MFI. Data are presented as mean ± SEM from n = 3 independent experiments. Statistical significance was determined using one-way ANOVA followed by Tukey's multiple-comparison test.

**Figure S8. Individual fluorescence channels and merged images of complement activation products and lysosomes.** (**A**) Representative confocal images showing the individual channels for nuclei (blue), C3b (red), and LAMP2 (green), together with the corresponding merged images. These images are reproduced from **Fig.4** and **Fig.5**. (**B**) Representative confocal images showing the individual channels for nuclei (blue), C5b9 (red), and LAMP2 (green), together with the corresponding merged images. These images are reproduced from **Fig.4** and **Fig.5**.

**Figure S9. Upstream C3 inhibition comprehensively inhibits the formation of C3a, C3b, C5a and MAC**. A2E-laden ARPE-19 cells were treated with 40% NHS in the presence of either the C3-Nf (40 µM) or the C5-DNb (20 µM). HI-NHS served as the control. (**A, C**) Level of anaphylatoxins (**A**) C3a and (**C**) C5a in the cell culture supernatant, measured by ELISA and quantified as OD450. (**B, D**) Deposition of complement fragments (**B**) C3b and (**D**) C5b9 on the RPE cell surface, measured by flow cytometry and quantified as MFI. Data are presented as normalized relative values calculated as: Relative Value (%) = [(Sample − Min) / (Max − Min)] × 100. Data are presented as mean ± SEM from n=3 independent experiments. Statistical analyses were performed using GraphPad Prism 9 (https://www.graphpad.com) by one-way ANOVA with Tukey's multiple comparison test. ∗∗∗∗=p < 0.0001, ∗∗∗=p < 0.001, ∗∗=p < 0.01, ∗=p < 0.05, ns=non-significant.

**Figure S10**. SDS-PAGE analysis of purified recombinant C3-Nf and C5-DNb.

**Reference**

1.	Chen, Z., et al., *Modulating the complement system through epitope-specific inhibition by complement C3 inhibitors.* Journal of Biological Chemistry, 2025. **301**(3).
